# Innovation of Critical Bubble Electrospinning and Its Mechanism

**DOI:** 10.3390/polym12020304

**Published:** 2020-02-03

**Authors:** Ya Li, Aixue Dong, Jihuan He

**Affiliations:** 1 College of Textile Science and Engineering (International College of Silk), Zhejiang Sci-Tech University, 928 Erhao Dajie Road, Hangzhou 310018, China; 2 Key Laboratory of Clean Dyeing and Finishing Technology of Zhejiang Province, Shaoxing University, Shaoxing, Zhejiang 312000, China; aixue_dong@126.com; 3 National Engineering Laboratory for Modern Silk, College of Textile and Clothing Engineering, Soochow University, 199 Ren-Ai Road, Suzhou 215123, China

**Keywords:** critical bubble electrospinning, polymer, nanofiber, mechanism, jet

## 1. Introduction

Along with the advent of an ever-increasing demand for the nano-industrialization, nanofibers become a unique class with many fascinating properties due to their nanoscale diameters and high surface area to volume ratio [1]. Electrospinning has been recognized as a versatile and effective technique for the preparation of various functional nanofibers, which can be modified to gain enormous attention for end applications, such as energy storage, sensors, filtration, textiles, tissue scaffolds, biomaterials, smart adhesion and so on [2,3,4,5,6,7,8,9,10,11,12]. Nevertheless, manufacturing upscaling of nanofibers from the laboratory to industry is still limited by its low production capacity. Many efforts have thus been concentrated on modifying the process in order to facilitate its transition, among which one way to achieve commercially feasible, larger production volumes is to incorporate multi-emitter systems instead of a single needle [2,13]. Although the output has been obviously enhanced by this approach, how to avoid interactions between each jet during the electrospinning process and how to clean the jets after they stop working are still two key issues remained to impede the commercialization [14]. Subsequently, based on an observation that jet number rises up even from free and nearly flat liquid surfaces, the needleless electrospinning that is of theoretical and practical interest starts to come into being for generation of multiple jets on a large scale [15]. It appears to have immense potential in the development of associated nanotechnologies and comprehensive understanding of some yet unknown mysteries of nature [15,16].

Emitters in needleless electrospinning play crucial roles in determining the process, fiber quality and productivity [17]. They can be classified into two categories according to their working states: rotating or stationary emitters, the former can introduce mechanical vibration to the polymer solution for assisting in initiating jets continuously, and the latter often need auxiliary force (e.g., magnetic field, gravity and gas bubble) to initiate the process [18]. The bubble electrospinning was first invented with an intrinsic property that used an electrostatic force to overcome the surface tension of polymeric bubbles, but it was very difficult to control the bubble’s size and number on a polymer solution surface [19]. The surface charges of both merged parts of the surfaces will become a common wall to minimize their surface area when two bubbles meet together, then the smaller bubble bulges into the larger bubble, it is not an advantage for generating jets during the bubble electrospinning process [20]. The minimal surface can be determined by the variational theory [21,22,23,24,25,26]. The number of produced bubbles reduces remarkably on the gas tube, only a single bubble is formed when its diameter decreases to a threshold value [27,28]. Ren et al. reduced the tube’s diameter to use a single bubble electrospinning system to overcome the shortcoming of merged bubbles, where only one single bubble was produced [27].

However, there is no essential mechanism difference between the two conventional bubble electrospinning methods mentioned above. When an electric field is presented, the charges in the bubble surface are induced and then relaxed quickly, so the coupling of surface charges and the external electric field will result in the deformation of bubbles into protuberance-induced upward-directed reentrant jets [20]. The bubble will burst once the electric field exceeds the critical value needed to overcome the surface tension, the electronic force pulls the bubble fragments upwards to generate jets. Owing to massive energy loss at the moment of bubble rupture, more stable spinning bubbles need to be considered. It is well known that the applied voltage in electrospinning has an important influence on resultant fiber characteristics, and the higher voltage and lower surface tension can improve the fiber quality [29,30,31]. Meanwhile, an important tribute of the bubble is that its surface tension reduction only relates to its radius, the inside and outside pressure, respectively [32].

In this paper, inspired by the described ideas and comprehensively considering the practical problems, the breakthrough was found from the connections between them. Only when a single bubble was guaranteed to be independent and stable, good performance of each bubble could be completely realized. To optimize the independent bubble electrospinning process parameters and control its stability and size, a new method, critical bubble spinning, was proposed, whose continuous spinning behavior could be achieved by the disturbance of the bubble surface from the steady liquid film in airflow and electric fields. Subsequently, innovation of critical bubble electrospinning was carried out to study its mechanism.

## 2. Experimental and Discussion

### 2.1. Experimental Setup

**Figure 1:**
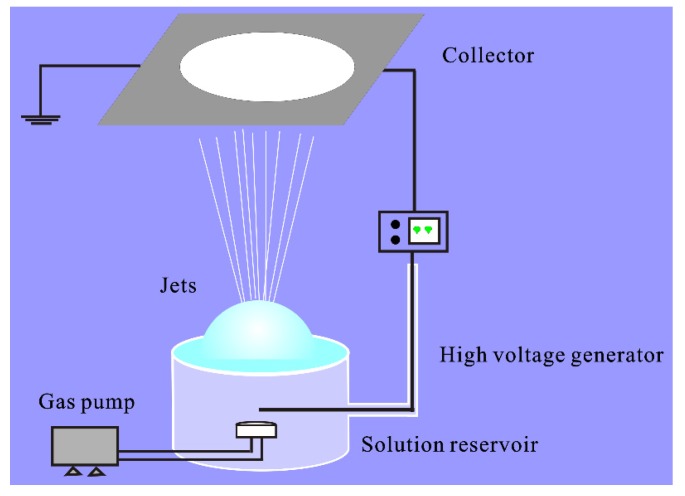
Schematic illustration of the critical bubble electrospinning.

The schematic illustration of the experimental setup used in this study was shown in Figure 1, which included a solution reservoir as the bubble generator, a gas pump, a high-voltage power supply and a grounded collector. Bubbles were produced by a controllable syringe pump, and a single bubble was stably produced in the aim of studying the effect of various spinning parameters. The bubble could keep stable for a relatively only time under the critical condition when internal and external force fields were balanced before its blasting. The solution level was guaranteed to be higher than the gas nozzle, and the flow of gas must be controlled at the appropriate scale. If the gas’ flow rate was too high, the bubbles could not be formed or blasted instantaneously, and multiple bubbles would be generated if it was too small.

### 2.2. Materials

Polyvinyl Alcohol (PVA1788), Polyether sulfone (PES), Polyvinylpyrrolidone (PVP k30), *N*,*N*-dimethylformamide (DMF) and N,N-dimethylacetamide (DMAC) were purchased from Sinopharm Chemical Reagent Co., Ltd. (Shanghai, China). 4,4′-Oxydianiline (ODA), pyromellitic anhydride (PMDA), zirconyl chloride octahydrate (ZrOCl_2_·8H_2_O, ≥98%) and ethanol (99%) were purchased from Sigma-Aldrich (St. Louis, MO, USA). All chemicals were of analytical grade and used without further purification.

### 2.3. Preparation of Different Polymer Solutions and Nanofibers

All concentration measurements were done in weight by weight. PVA aqueous solutions were prepared with 6, 7 and 8 wt %, respectively. Then the solutions were gently stirred at 90 °C for two hours. PES/DMF and PVP/distilled solutions were prepared with 28 and 30 wt %, respectively. The precursor solution was prepared by adding ZrOCl_2_·8H_2_O in ethanol dropwise to an 8 wt % PAN solution in DMF, the molar ratio of DMF and ethanol was controlled at 20:1 and the weight ratio of ZrOCl_2_·8H_2_O and PAN was 3:2. ODA was introduced to a three-necked bottle containing DMAC and stirred for 20 min under mechanical stirring. Then PMDA was added to the above solution to make the concentration at 24 wt % by three times within 1 h in an ice bath and stirred vigorously for 3 h to produce the PAA solution.

In the critical bubble electrospinning experiments, a high electric potential of 27 kV and a distance between the bubble top and the collector of 25 cm were applied to PVA spinning processes. The applied voltage was set at 35 kV and the collecting distance was kept as 25 cm for PES nanofibers. Similarly, as to PVP nanofibers, the applied voltage was set at 30 kV and the bubble top to the collector distance was set as 25 cm. The PAN/ZrCl_2_·8H_2_O/DMF/ethanol precursor solution set on the critical bubble electrospinning setup was applied a voltage of 25 kV for nanofiber formation with a collecting distance of 25 cm. The as-spun nanofibers were sintered at 1000 °C for 2 h in air with a heating rate of 5 °C min^−1^, in which ZrOCl_2_·8H_2_O was hydrolyzed to form inorganic ZrO_2_ while PAN was burned up [33,34]. The PAA as-spun nanofibers were obtained at a voltage of 28 kV and the distance between the bubble top and collector was 25 cm, the mat was stabilized at 80 °C for 2 h in air with a heating rate of 2 °C min^−1^, which were then heated at 160 and 250 °C for 1 h with a heating rate of 2 °C min^−1^, respectively, and at last both heated at 300 and 300 °C for 30 min to get polyimide (PI) nanofibers.

The parameters, such as concentration, voltage and collecting distance were the same for the critical bubble electrospinning and single-bubble electrospinning, while for the single-needle electrospinning, 7 wt % PVA, 25 wt % PES, 26 wt % PVP and 16 wt % PAA were fabricated at 20 kV and 15 cm, respectively, and the voltage was set at 16 kV and the collecting distance was 15 cm for 7 wt % PAN/ZrCl_2_·8H_2_O/DMF/ethanol solution. All calcination conditions were the same for the three spinning methods in regards to the precursors. The ambient relative humidity was 40% ± 2% and temperature was 25 ± 2 °C and maintained constants. The diameters of nanofibers were calculated by measuring nanofibers at random using Image J software.

### 2.4. The Theoretical Model of the Critical Bubble

According to the Young–Laplace equation [35,36], the surface tension of a hemispheric bubble made by the aqueous fibroin solution could be expressed as:

(1) $$2\pi r\sigma =\pi r^{2}\left(P_{i}-P_{o}\right)$$

**Figure 2:**
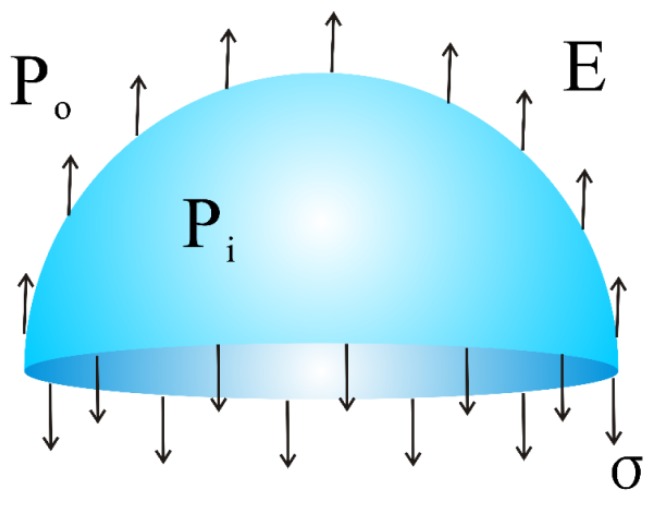
The force analysis model of the bubble during critical bubble electrospinning.

Therefore,

(2) $$\sigma =\frac{1}{2}r\left(P_{i}-P_{o}\right)$$

where σ was the surface tension, r the radius of the bubble, $P_{i}$ and $P_{o}$ the air pressures inside and outside the bubble, respectively [37,38]. During critical bubble electrospinning, considering the forces applied to a bubble (see Figure 2) generated from the surface of the polymer solution, the theoretical model of its process parameters could be obtained [33]. By the assumption of balance, the force pushing the hemispheric bubble upward was expressed in the form:

(3) $$F_{upward}=2\pi r^{2}qE+\pi r^{2}\left(P_{i}-P_{o}\right)$$

In addition, the force downward around the circle was

(4) $$F_{download}=2\pi r\sigma$$

This gave

(5) $$2\pi r^{2}qE+\pi r^{2}\left(P_{i}-P_{o}\right)=2\pi r\sigma$$

among which q was the charge per unit area and E the applied electric field.

From Equation (5), we could immediately obtain:

(6) $$2rqE+r\left(P_{i}-P_{o}\right)=2\sigma$$

Equation (6) showed the surface tension of the polymer bubble depended mainly on the bubble size, pressure difference between the inside and outside of the bubble, and the electronic field. When the electronic force was high enough to overcome the surface tension, the bubble would be broken.

According to the Bernoulli equation, we had

(7) $$\frac{P_{i}}{\rho_{i}}+\frac{1}{2}u^{2}=B,\;\frac{P_{o}}{\rho_{o}}+\frac{1}{2}u^{2}=B\;$$

(8) $$\;2rqE+r\left(BP_{i}-\frac{1}{2}\rho_{i}u^{2}-P_{o}\right)=2\sigma \;$$

Combining it with the conservation of the mass equation

(9) $$Q=A\rho_{i}u$$

where u was the velocity, $\rho_{i}$ and $\rho_{o}$ are the densities of the air inside and outside of the bubble, A the section area and Q the flow rate.

Then we could get

(10) $$2rqE+r\left(BP_{i}-\frac{1}{2}\rho_{i}{\left(\frac{Q}{A\rho_{i}}\right)}^{2}-P_{o}\right)=2\sigma$$

Based on Equation (10), assuming E was a constant, we could obtain

(11) $$2qEr+r\left(BP_{i}-\frac{1}{2}\rho_{i}{\left(\frac{1}{A\rho_{i}}\right)}^{2}Q^{2}-P_{o}\right)=\sigma$$

It meant that when the critical equilibrium state was controlled at the constant voltage, the relationship between the bubble size and the incoming gas could be expressed as

(12) $$r=\frac{\sigma }{2qE+B\rho_{i}-\frac{1}{2}\rho_{i}{\left(\frac{1}{A\rho_{i}}\right)}^{2}Q^{2}-P_{o}}$$

**Figure 3:**
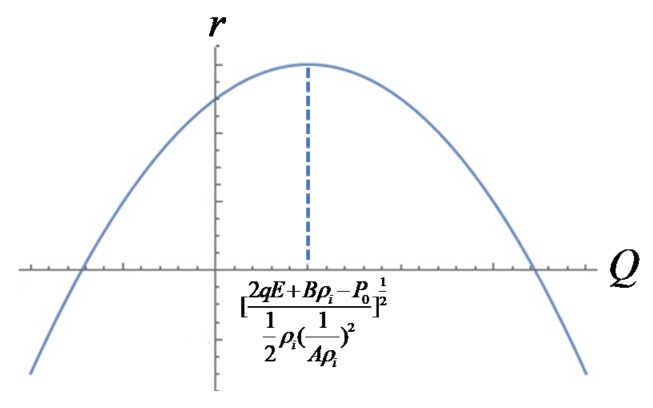
Diagram of the function between r and *Q.*

Therefore r was the biggest when *Q* reached $\;{\left[\frac{2qE+B\rho_{i}-P_{0}}{\frac{1}{2}\rho_{i}{\left(\frac{1}{A\rho_{i}}\right)}^{2}}\right]}^{\frac{1}{2}}$ as illustrated in Figure 3.

Similarly, if r was a constant, then the following relational expression could be obtained

(13) $$2qEr+r\left(B\rho_{i}-P_{o}\right)-\frac{1}{2}r\rho_{i}{\left(\frac{1}{A\rho_{i}}\right)}^{2}Q^{2}=\sigma$$

So, when the critical bubble was controlled at the same size, the relationship between the applied electric field and the incoming gas would be

(14) $$E=\frac{\sigma -r\left(B\rho_{i}-P_{o}\right)+\frac{1}{2}\rho_{i}{\left(\frac{1}{A\rho_{i}}\right)}^{2}Q^{2}}{2qr}$$

**Figure 4:**
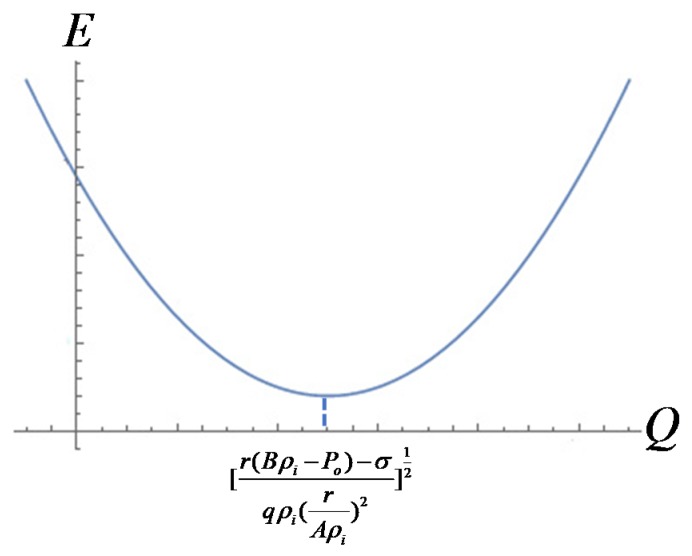
Diagram of the function between E and Q .

Hence, the electric field needed the weakest when *Q* reached ${\left[\frac{r\left(B\rho_{i}-P_{o}\right)-\sigma }{q\rho_{i}{\left(\frac{r}{A\rho_{i}}\right)}^{2}}\right]}^{\frac{1}{2}}\;$, just as shown in Figure 4, the electric field became the weakest.

What is more, as Q was a constant value, according to Equation (10), that meant

(15) $$2qEr+rB\rho_{i}-\frac{1}{2}r\rho_{i}{\left(\frac{Q}{A\rho_{i}}\right)}^{2}-P_{o}=\sigma$$

The flow rate remained the same, the relationship between the applied electric field and the incoming gas could be expressed in the form

(16) $$r=\frac{\sigma }{2qE+B\rho_{i}-\frac{1}{2}\rho_{i}{\left(\frac{Q}{A\rho_{i}}\right)}^{2}-P}$$

**Figure 5:**
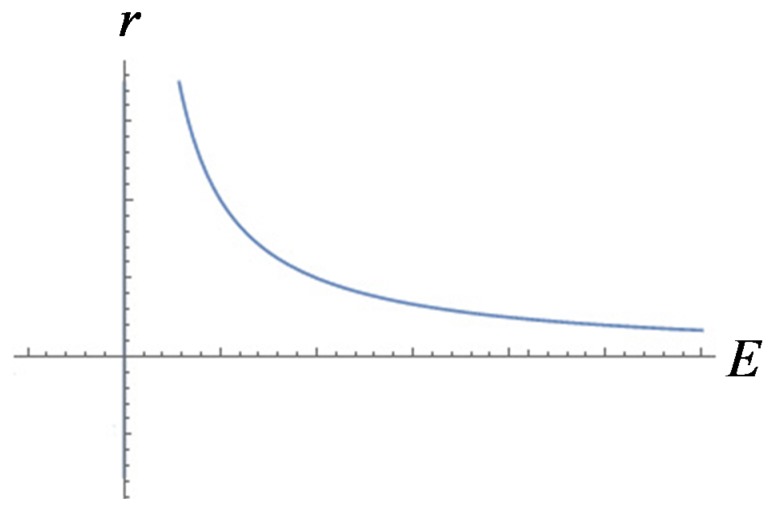
Diagram of the function between E and r .

It should be noticed that the electric field was inversely proportional to the radius of the bubble as shown in Figure 5.

### 2.5. Instability of the Bubble Surface Under the Critical Bubble Electrospinning Process

A stable bubble was of great importance in the critical bubble electrospinning process, however, the instability of the bubble surface would greatly affect the jet number. The instability of the bubble surface in the critical bubble electrospinning process was seen to be transferred along the bubble surface from the solution surface to the jets, and more and more micro or nano scale bubbles were formed on the bubble surface, as a result, more and more jets were produced, and more and more solutions would be transferred along the bubble wall. When the solution was not enough to be transferred to each jet, the bubble would be soon broken, and spinning process would be stopped until another critical bubble was produced.

**Figure 6:**
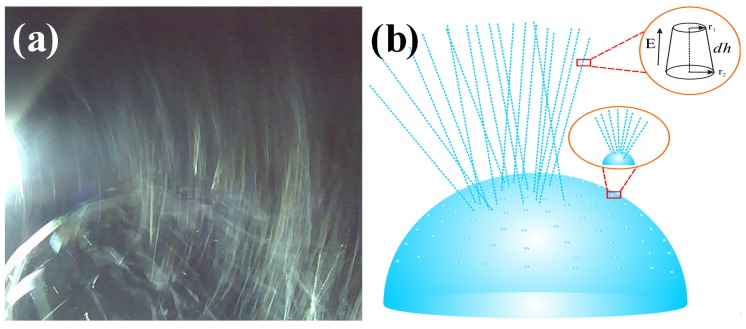
(**a**) Jet formation and (**b**) its mechanism in critical bubble electrospinning (7 wt % PVA solution).

**Figure 7:**
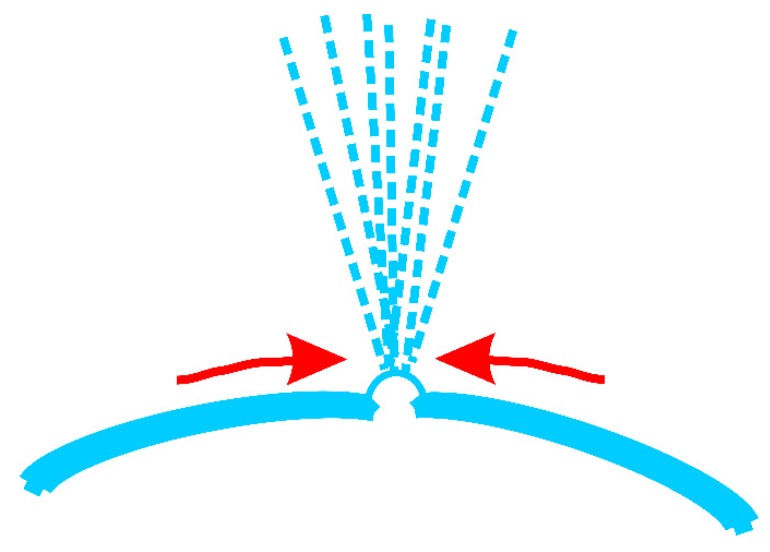
The flow around a sub-daughter bubble during the generating jets process.

Multiple jets, looking like a waterfall, produced from critical bubble electrospinning were illustrated in Figure 6a. Both experimental and numerical studies revealed that once the electric field exceeded the critical value needed to overcome the surface tension, the bubble was broken, and the curved film of the ruptured bubble would fold and entrap air as it retracted; the resulting toroidal geometry of the trapped air was unstable, leading to the creation of a ring of smaller bubbles [39,40]. According to the principle of mass conservation, when the energy was exhausted, shown in Figure 6b, numerous smaller daughter bubbles were formed, which were then further pulled upwards by the forces, and the daughter bubbles were broken again to form sub-daughter bubble cascades in sequence. The sub-daughter bubbles were mini, their intrinsic stabilization time was greatly shortened. The Laplace pressure inside the sub-daughter bubble dimpled the interface to create a cavity, but the film rapidly healed itself because of the flow direction around, see Figure 7. The process continued until some hierarchical ruptured bubble was pulled upwards to form a charged jet, ejecting into the metal receiver as nanofibers.

Therefore, the entire critical bubble electrospinning process was implemented by countless daughter bubbles, and the dynamic behavior of each daughter bubble was the same as that of the original one. Relatively speaking, the required surface energy was small, so the continuity and stability of the process were realized, which could guarantee the quality and yield of nanofibers prepared.

**Figure 8:**
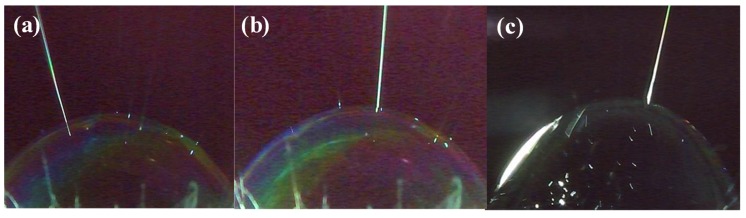
Apical advantage in critical bubble electrospinning (7 wt % PVA solution): (**a**) top left, (**b**) almost centered, (**c**) top right.

**Figure 9:**
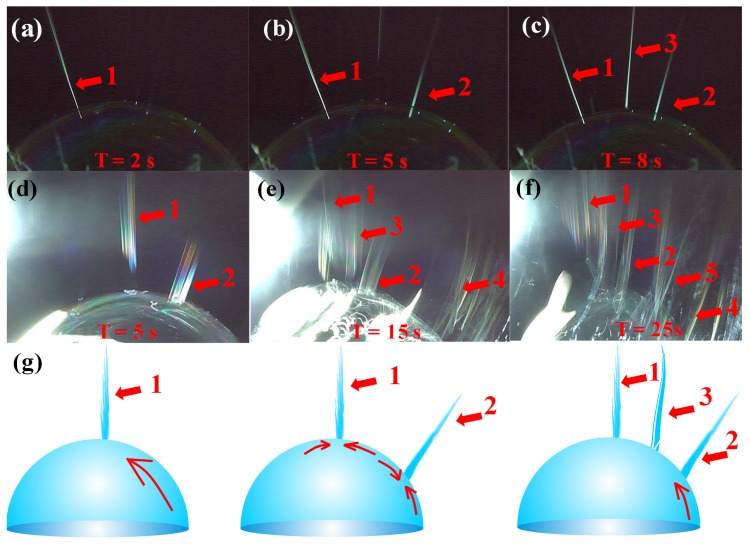
The law of jets formation in critical bubble electrospinning: images of (**a**) the first jet , (**b**) the second jet and (**c**) the third jet generated for 6 wt % PVA solution; images of (**d**) the first jet , (**e**) the second jet and (**f**) the third jet generated for 8 wt % PVA solution; (**g**) illustrations of the “array in two points” law.

Owing to the external forces, the first jet was concentrated at the apical position (Figure 8a–c). Once a new jet was created due to the surface disturbance of the liquid film at a different position, then the former state was broken, new jets were generated one by one between the two places, just as the numerical order in Figure 9a–f. Figure 9a–c showed the jet generation for 6 wt % PVA solution and Figure 9d–f for 8 wt % PVA solution, respectively. Thus, more and more jets were produced in accordance with the law of “array in two points” as illustrated in Figure 9g. The continuously kept time for the bubble in the critical state lasted about 30 s.

### 2.6. Continuous Spinning Process

**Figure 10:**
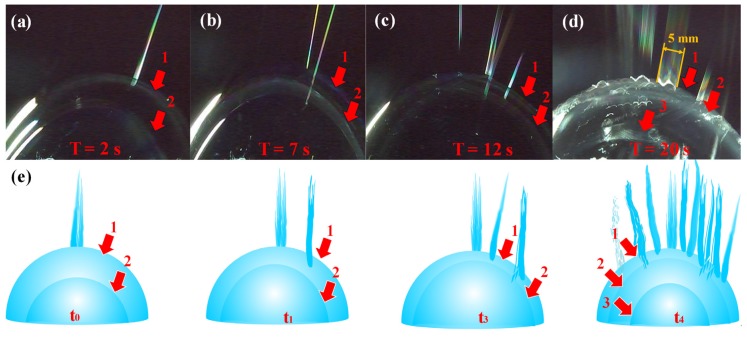
The growth process of bubbles in critical bubble electrospinning: images of continuous bubbles at (**a**) 2s, (**b**) 7s, (**c**) 12s, (**d**) 20s; (**e**) illustrations of the continuously growing bubbles and generated jets.

The continuity of critical bubble electrospinning was realized by the bubbles growing one by one, such as bubble 1, 2 and 3 (Figure 10a–e). As shown in Figure 10d, the width of the jets was approximately 5 mm from the bubble whose diameter was about 60 mm. Whereas the mean diameter of the nanofibers prepared by this critical bubble was nearly 300 nm, there were as many as 16,667 ($\frac{5\;mm\left(the\;single\;line\;length\right)}{300\;nm\left(the\;mean\;diameter\;of\;the\;nanofibers\;obtained\right)}\;$) nanofibers arranged in a single line, meaning that the steady critical electrospinning process was highly efficient. Since a higher number of multiple jets implied a higher efficiency of electrospinning process, the spinning process would be perfect for producing a maximal number of the multiple jets.

**Figure 11:**
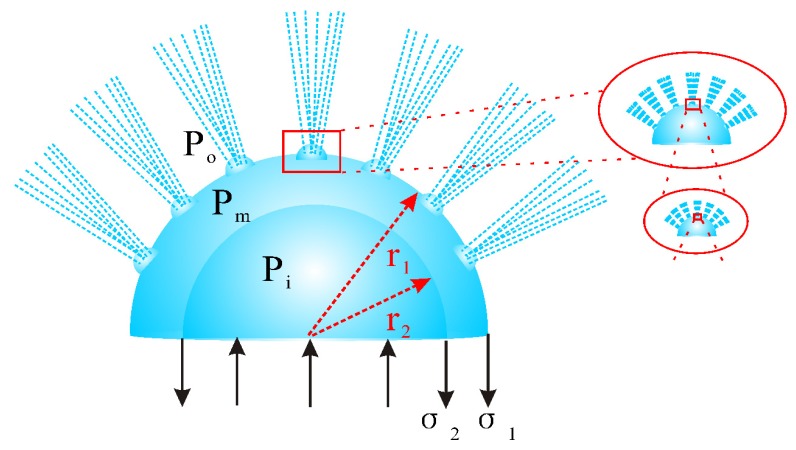
Schematic of hierarchical bubbles in critical bubble electrospinning.

In Figure 11, it could be seen that there were outside and inside bubbles with the diameters of $r_{1}$ and $r_{2}$, the surface tension of $\sigma_{1}$ and $\sigma_{2}$, the outside, middle and inside air pressures of $P_{o}$, $P_{m}$ and $P_{i}$, respectively. According to the experimental observation and the balance force analysis, theoretical models of continuous spinning process were established as follows:

(17) $$\pi r_{1}{}^{2}\left(P_{m}-P_{o}\right)+E=2\pi r_{1}\sigma_{1}$$

(18) $$\pi r_{2}{}^{2}\left(P_{i}-P_{m}\right)=2\pi r_{2}\sigma_{2}$$

Owing to the existence of the hierarchical bubbles in critical bubble electrospinning, the continuous spinning process could go on consistently.

### 2.7. Nanofibers Fabricated by Critical Bubble Electrospinning

**Figure 12:**
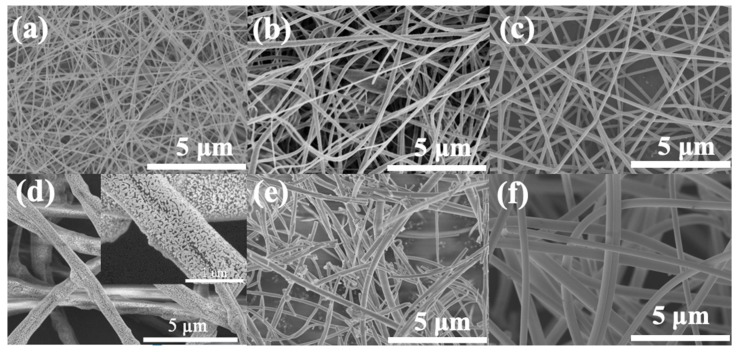
Nanofibers prepared by critical bubble electrospinning, (**a**) 7 wt % PVA/aqueous solution (mean diameter: about 114 nm), (**b**) 28 wt % PES/DMF (mean diameter: about 187 nm), (**c**) 30 wt % PVP/distilled water/ethanol (mean diameter: about 146 nm), (**d**) 8 wt % PAN/ZrCl_2_·8H_2_O/DMF/ethanol (mean diameter: about 884 nm) and (**e**) its product obtained after calcination at 1000 °C in air (mean diameter: about 226 nm) and (**f**) PI nanofibers from 24% ODA/DMAC/PMDA precursor (mean diameter: about 432 nm).

**Figure 13:**
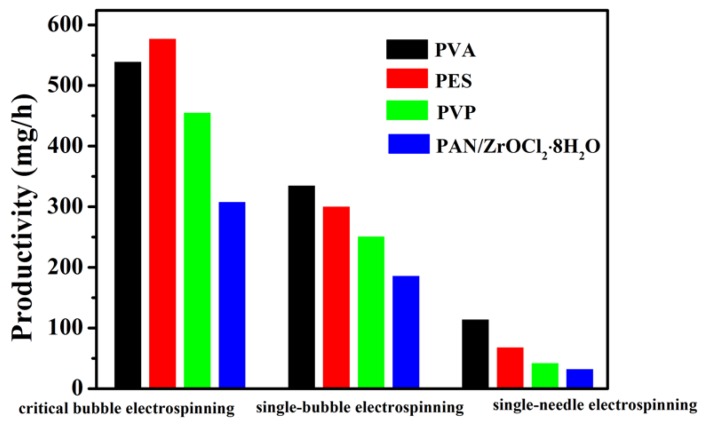
The nanofiber productivity of single-needle/bubble electrospinning and critical bubble electrospinning.

In a number of publications, efforts have been described in finding the controlling parameters for different polymers using bubble electrospinning [41,42,43,44,45,46,47,48,49]. The morphologies of various nanofibers obtained using critical bubble electrospinning were carried out by SEM, respectively, which were shown in Figure 12. It should be noted that not only organic but also inorganic [34] high-quality nanofibers could be fabricated successfully via the modified bubble electrospinning method. Therefore, the universality of critical bubble electrospinning was verified, indicating that the diameter of nanofibers prepared by critical bubble electrospinning was within the controllable range and had operational stability. At the same time, the same solution was prepared by a traditional single-needle/bubble electrospinning technique to compare the yield with that from critical bubble electrospinning, as shown in Figure 13. It should be seen that the proposed critical bubble electrospinning could significantly increase the yield of nanofibers.

## 3. Conclusions

To summarize, an innovative approach, called critical bubble electrospinning, taking advantage of a single independent and stable bubble, was presented successfully to obtain high throughput preparation of nanofibers. Nanofibers were electrospun from numerous perturbations located at the free surface of the steady polymer bubble film based on the combination of normal airflow and electric fields. Subsequently, critical bubble electrospinning was deeply analyzed to achieve the critical forces, a theoretical model was established and the process of generating jets was recorded with a high-speed camera to study the formation law of multiple jets. Therefore, the entire critical bubble electrospinning process was implemented by countless daughter bubbles, and the required energy was small, the continuity and stability of the process were realized, which could guarantee the quality and yield of nanofibers prepared. Finally, the universality of critical bubble electrospinning was verified to be improved, concomitantly promoting the feasibility of practical applications. Therefore, critical bubble electrospinning had some innate advantages in electrospinning and its set-up also deserved further exploration on the improvement and optimizing.
